# Maturity impact on physicochemical composition and polyphenol properties of extra virgin olive oils obtained from *Manzanilla*, *Arbequina*, and *Koroneiki* varieties in Iran

**DOI:** 10.1002/fsn3.3497

**Published:** 2023-06-16

**Authors:** Seyed Amirreza Ghreishi Rad, Maryam Jalili, Farzaneh Ansari, Hamid Rashidi Nodeh, Ladan Rashidi

**Affiliations:** ^1^ Department of Mechanical Engineering of Biosystems, Faculty of Agriculture Engineering and Technology, College of Agriculture and Natural Resource Tehran University Tehran Iran; ^2^ Research Center of Food Technology and Agricultural Products Standard Research Institute of Iran (SRI) Karaj Iran

**Keywords:** extra virgin, maturation, olive oil, olive oil chromatography

## Abstract

This study investigated the physicochemical properties and polyphenol composition of extra virgin olive oils (EVOOs) extracted from three olive cultivars. The investigated cultivars were *Arbequina*, *Koroneiki*, and *Manzanilla*, grown in Olive Research Station in Rudbar county, Gilan province, Iran, at three ripening stages. Several parameters were analyzed, including peroxide and acidity values, unsaponifiable matter, oxidative stability, total aliphatic alcohols, fatty acids (FAs), sterols, and triacylglycerol composition. The results showed that as maturity increased, parameters such as oil content, acidity value, and iodine value, rise, while parameters including peroxide value, oxidative stability, aliphatic alcohols, and unsaponifiable matter decreased (*p* < .05). The saponification value was slightly reduced in the developing ripening process (*p* > .05). The MUFA/PUFA ratio and total sterol content declined during the olive ripening stages (*p* < .05). The triterpenes decreased in *Arbequina* and *Koroneiki* cultivars but increased in *Manzanilla* cultivar during the maturity stages. According to the data, oleuropein decreased while oleuropein aglycone, oxidized aldehyde, and hydroxylic form of oleuropein increased for all EVOOs during maturation. Apigenin, quercetin, ligstroside aglycone, aldehyde and hydroxylic form, ferulic acid, caffeic acid, and catechin decreased during the ripening of fruits (*p* < .05). The main triglycerides were triolein (OOO), palmitodiolein (POO), dioleolinolein (OOL), and palmitooleolinolein (PLO) in all EVOOs. In addition, the olive cultivar and harvesting date influence the physicochemical properties and polyphenol composition of EVOOs extracted from olive varieties grown in one region. In conclusion, the results can present helpful information to determine the optimum maturity stage for the investigated olive cultivars.

## Graphical abstract

The present study provides information about the maturation process of three cultivars, including Arbequina, Koroneiki, Manzanilla cultivated in Rudbar county, Gilan province. This research has evidenced that the maturation advances influence significantly the chemical composition and polyphenol contents of extracted EVOOs. Therefore, the determination of these characteristics can be applied to specifying the optimum time for olive fruit harvesting of a specified variety.

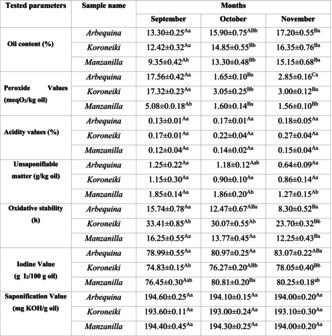

## 1 INTRODUCTION

Extra virgin olive oil (EVOO), extracted from healthy olive fruits by mechanical procedure, has extraordinary health benefits in preventing chronic disease. The meta‐analysis studies of the Mediterranean diet and olive oil consumption show the risk reduction of cardiovascular diseases, atherosclerosis, and particular types of cancer (Fratianni et al., 2019; Khadem et al., 2019; Pourghorban et al., 2022). Olive oil comprises 98% triacylglycerols and minor fat‐accompanying compounds, including aliphatic alcohols, tocopherols, phenolic compounds, and phytosterols. In many studies, high‐quality EVOO is considered a natural pharm‐food. These health benefits of EVOO are due to its fat composition, including high oleic acid concentration (56–84%), linoleic acid (3.5–21%), and linolenic acid lower than 1.5%, and the presence of bioactive compounds such as β‐carotene, tocopherols (vitamin E), volatile compounds, sterols, and phenolic compounds (PCs) (Lanza & Ninfali, 2020). The bioactive compounds of olive fruits may be affected by several factors, including growing season, variety, temperature, soil type, light, growing environment, and processing, and post‐harvest storage (Alfaro et al., 2013). Optimization of harvesting time will benefit the olive industry dramatically. The early harvest of healthy olive fruits results in EVOOs with higher nutritional value and sensory properties attributed to the high concentration of polyphenols. In early harvest, olive fruits have low oil content and unique sensory properties, including high bitterness and excess pungency, but the late harvest of olive fruits possess high oil content with less quality and show lower sensory characteristics (Dag et al., 2021).

The physicochemical composition and polyphenol content of EVOOs depend on the environmental growth conditions, including biotic and abiotic stresses, the genetic background, and the agronomic techniques (Conde et al., 2008). When the fruits ripen, the pulp and the peel color change from deep green to black, and olive oil's organoleptic properties change dramatically (Franco et al., 2015).

In addition, maturity index (MI) depends highly on the environmental growth conditions, including biotic and abiotic stresses, the genetic background, crop load, cultivation practices, and agronomic techniques (Kafkaletou et al., 2021; Touati et al., 2022).

According to the International Olive Council, Iran is the 17th country in olive production, and 92,000 hectares are under olive cultivation in Iran. Olive trees are cultivated in 26 out of 31 provinces of Iran, most of which are produced in the north of Iran. About 60% of the cultivars are natives, and 40% are foreign. Typically, the harvesting season of olive fruits in Iran starts at the end of September and lasts approximately 15 days, which depends on the weather conditions and the season. Manual harvesting is performed by 3 to 4 people per olive tree.

This study investigated the physicochemical and bioactive compound characteristics of three EVOOs from different foreign varieties cultivated in north Iran.

## 2 MATERIALS AND METHODS

### 2.1 Materials

Methanol, 99.99%, n‐hexane, 99.99%, orthophosphoric acid, 85%, and acetonitrile, 99.9%, with chromatography grade, were purchased from Fisher Scientific (Lisbon, Portugal). Folin‐Ciocalteu thiosulfate and 2, 2‐Diphenyl‐1‐picrylhydrazyl (DPPH) reagents, standards of phytosterols, fatty acid methyl esters, triglycerides, vanillic acid, vanillin, caffeic acid, oleuropein, tyrosol, cinnamic acid, luteolin, catechin, gallic acid, apigenin, and ferulic acid were purchased from Sigma‐Aldrich (USA). Other materials, including acetonitrile, acetone, and diethyl ether, were obtained from the Merck Company (Germany).

### 2.2 Samples preparation

The research was performed during the 2021 olive harvest season. Foreign cultivars of “*Arbequina*, *Manzanilla*, and *Koroneiki*” were harvested in Olive Research Station, Rudbar County, Gilan province, Iran. The geographical coordinates of the Olive Research Station were 36°48′N 49°24′E. This orchard is 1050 meters above sea level. Olive fruits were harvested by hand from five trees at various harvesting dates (1–3 kg), and based on fruit skin color, the ripening stages were categorized into green, black, green, and purple with uniform characteristics. The samples were prepared for three consecutive months in September, October, and November. The fruits were washed with water, and their pulp was separated from whole stones. Then, olive oil was extracted by cold pressed processing immediately after the preparation of samples (for each sample, 1 kg) by laboratories mill, Abencor® system. The temperature of the extraction procedure was maintained below 40°C. The extracted oil was stored in a cold and dark room prior to further analysis.

### 2.3 Oil recovery

The oil content of each variety was determined as the percentage of fresh olive paste by the following equation:

$$\text{Olive oil yield}=\left(V\times D/W\right)\times 100$$

Where *V* is the volume of EVVO (mL); *D* is the density of EVOO (0.915 g mL^−1^); and W is the weight of olive paste (Yorulmaz et al., 2013).

### 2.4 Determination of quality parameters and physicochemical characteristics

#### 2.4.1 Peroxide value, acidity value, unsaponifiable matter, oxidative stability

Peroxide value, acidity value, unsaponifiable matter, and oxidative stability were determined according to the methods described in International Organization for Standardization (ISO) 3960 (2017), 660 (2020), 18609 (2000), and 6886 (2016), respectively.

##### Determination of peroxide value

According to ISO 3960, 5 g of olive oil sample was weighed and mixed with 50 mL of a solution containing isooctane: acetic acid (60:40). Then 0.5 mL of saturated KI solution was mixed and kept in the dark place. After 1 min, 100 mL of distilled water was mixed, and the final solution was titrated with Na_2_S_2_O_3_ solution (0.01 N) until the appearance of light color (indicator was 1% starch solution). The peroxide value was calculated by the formula (1):

$$\mathrm{PV}\;\left(\mathrm{meq}\;O_{2}/\mathrm{kg}\;\text{oil}\right)=\left(V_{s}–V_{b}\right)\times F\times N\times 1000/W$$

*V*_s_ is the titration volume of the sample (mL); *V*_b_ is the titration volume of blank (mL); *F* is Na_2_S_2_O_3_ (0.01 N) factor; *W* is the weight of oil in volume (g); and *N* is the normality of Na_2_S_2_O_3_ solution (ISO 3960, 2017).

##### Determination of acidity value

The volumetric titration method determined the acidity value (ISO 660, 2020). For measuring the acidity value, the olive oil was dissolved in hot, neutral ethanol (100 mL) and titrated with NaOH (0.1 N). The acidity value was obtained by formula (2):

FFA=0.5×56.1×c×v/m

*c* is the concentration of NaOH (mol/L); *v* is the volume of used NaOH (mL); 56.1 (g/mol) of potassium hydroxid and *m* is sample weight (g).

##### Determination of unsaponifiable matter

The unsaponifiable matter amount was measured by saponifying each sample using ethanolic potassium hydroxide solution and extraction of soap solution with diethyl ether. Diethyl ether was evaporated, and the residue was weighed after drying. The results were reported as the percentage of unsaponifiable matter (ISO 18609, 2000).

##### Determination of oxidative stability

Based on the described methodology in ISO 6886, the oxidative stability of samples was measured by the Rancimat method, which is an accelerated aging test. Olive oil (2.5 g) was weighed in the vessel and put in the thermal place at 110°C. The hot air was passed through the oil in the reaction vessel. Volatile secondary reaction products were transported by air flow (20 L/h) into the measuring container and absorbed by distilled water. The increase in electrical conductivity was recorded simultaneously due to the absorption of the ionic reaction products (ISO 6886, 2016).

##### Saponification value and iodine value

Saponification and iodine values were measured according to ISO 3657 (2020) and ISO 3961 (2018), respectively. According to ISO 3657, saponification value is based on measuring the free and esterified acids in edible fats, oils, and fatty acids. This reference method calculates saponification value from fatty acid data obtained by gas chromatography analysis. Also, ISO 3961 is a reference method for measuring the iodine value of animal and vegetable fats and oils using fatty acid composition data.

#### 2.4.2 Determination of fatty acids (FAs) composition

After purification of olive oils (if necessary), 0.1 g of the olive oil was weighed and mixed with 2 mL of heptane. Then 0.2 mL of methanolic potassium hydroxide solution was added and shaken for the 30 s. After 1 min, the clear upper layer containing methyl esters was injected into the gas chromatography coupled with a flame ionization detector (GC‐FID) (Yung Lin 6100, Korea). The column was CP‐Sil 88 (100 m × 0.25 mm × 0.25 μm), and the spilt ratio was set at 1:100. The carrier hydrogen gas flow rate was 1 mL/min. The injector and detector were adjusted at 250°C. The oven temperature program was 165°C (8 min) to 210°C at 2°C/min (COI/T.20/Doc. NO. 33, 2017).

#### 2.4.3 Determination of individual and total alcoholic content

The individual and total alcoholic content of olive oils were determined by COI/T.20/Doc. No 26 (2020) test method. 1‐eicosanol and α‐cholestanol as the internal standards were added to the sample oil (5 g) and saponified with ethanolic potassium hydroxide solution (50 mL, 2 M). In the next step, the unsaponifiable matter was extracted thrice with diethyl ether (70 mL). Lastly, the remaining solvent was evaporated. Then, the various alcoholic compound fractions were separated and marked using thin layer chromatography (TLC). Each fraction was recovered from silica gel, transformed into trimethyl ethers, and analyzed by GC‐FID equipped with the capillary column, DB‐5, 30 × 0.25 mm × 0.32 μm. The injector and detector temperatures were adjusted at 280 and 290°C, respectively. The oven program was 180°C (8 min) to 260°C (at 5°C/min) for aliphatic alcohols, and the oven temperature was adjusted to 260 ± 5°C (isothermal) for sterol composition. The split ratio was set at 1:50, and the flow rate of carrier hydrogen carrier gas was 1 mL/min.

#### 2.4.4 Determination of triacylglycerol composition

The composition of the experimental triacylglycerols (TAGs) was compared with theoretical data obtained from the analysis of fatty acid methyl esters (FAMEs). After oil purification by solid phase extraction (SPE) using a silica gel cartridge, the residues were dissolved in 1 mL of acetone and injected into HPLC. TAG composition was determined by HPLC (Yung Lin 9100, Korea) coupled with a refractive index detector. The HPLC column (25 cm × 4 mm) was packed with RP‐18 (4 μm particle size), and the mobile phase was propionitrile with a flowrate of 1 mL/min (COI/T.20/Doc. NO. 20, 2017). The theoretical TAG composition was calculated from the fatty acid composition using several mathematical algorithms described in the COI/T.20/Doc. No 20 (2018).

#### 2.4.5 Determination of phenolic compounds

Major phenolic compounds were identified and quantified according to the COI/T.20/DOC. 29 by an HPLC system (COI/T.20/Doc. NO 29, 2017). Briefly, 2 g of olive oil was weighed, and 1 mL of syringic acid (0.015 mg/mL) was added to it and mixed. 5 mL of the methanol/water (80:20) was added and mixed for 1 min. Then polyphenol compounds were extracted using an ultrasonic bath for 15 min. The ultrasonic bath temperature was maintained at 25°C by a thermometer. After centrifugation (10,000 rev/min, 10 min), 20 μL of supernatant was injected into HPLC coupled with a UV detector with C18 reverse‐phase column (4.6 mm × 25 cm, 5 μm) (COI/T.20/Doc. NO 29, 2017). Mobile phases were deionized water with 0.2% H_3_PO_4_ (A), methanol (B), and acetonitrile (C) which A 96%, B 2%, and C 2% (0–40 min), then A 50%, B 25%, and C 25% (40–45 min), then A 40%, B 30%, and C 30% (45–60 min), A 0%, B 50%, and C 50% (60–70 min). Polyphenol compounds were detected at 280 nm.

### 2.5 Statistical analysis

Statistical analyses were conducted using Minitab (Minitab Inc., PA, State College, USA) version 17.0. Comparison of the means was performed using ANOVA with post hoc Tukey's test at *p* < .05 (the confidence limit was based on 95%). All determinations were made in triplicate, and all data were demonstrated as mean ± SD.

## 3 RESULTS

### 3.1 Peroxide value, acidity value, unsaponifiable matter, oxidative stability, saponification, and iodine values

The results of the oil recovery percentage, peroxide value, acidity value, unsaponifiable matter, oxidative stability, iodine value, and saponification value in the EVOOs extracted from olive fruits of *Arbequina*, *Koroneiki*, and *Manzanilla* varieties, are shown in Table 1.

**TABLE 1:** Results of physicochemical properties determined in EVOOs, from the three olive varieties grown in the study area, collected at different stages of ripening, Data are presented as mean ± SD (*n* = 3).

Tested parameters	Sample name	Months		
		September	October	November
Oil content (%)	*Arbequina*	13.30 ± 0.25^Aa^	15.90 ± 0.75^ABb^	17.20 ± 0.55^Ba^
	*Koroneiki*	12.42 ± 0.32^Aa^	14.85 ± 0.55^Bb^	16.35 ± 0.76^Ba^
	*Manzanilla*	9.35 ± 0.42^Ab^	13.30 ± 0.48^Bb^	15.15 ± 0.68^Ba^
Peroxide values (meqO_2_/kg oil)	*Arbequina*	17.56 ± 0.42^Aa^	1.65 ± 0.10^Ba^	2.85 ± 0.16^Ca^
	*Koroneiki*	17.32 ± 0.23^Aa^	3.05 ± 0.25^Bb^	3.00 ± 0.12^Ba^
	*Manzanilla*	5.08 ± 0.0.18^Ab^	1.60 ± 0.14^Ba^	1.56 ± 0.10^Bb^
Acidity values (%)	*Arbequina*	0.13 ± 0.01^Aa^	0.17 ± 0.01^Aa^	0.18 ± 0.05^Aa^
	*Koroneiki*	0.17 ± 0.01^Aa^	0.22 ± 0.04^Aa^	0.27 ± 0.04^Aa^
	*Manzanilla*	0.12 ± 0.04^Aa^	0.14 ± 0.02^Aa^	0.15 ± 0.04^Aa^
Unsaponifiable matter (g/kg oil)	*Arbequina*	1.25 ± 0.22^Aa^	1.18 ± 0.12^Aab^	0.64 ± 0.09^Aa^
	*Koroneiki*	1.15 ± 0.30^Aa^	0.90 ± 0.10^Aa^	0.86 ± 0.14^Aa^
	*Manzanilla*	1.85 ± 0.14^Aa^	1.86 ± 0.20^Ab^	1.27 ± 0.15^Ab^
Oxidative stability (h)	*Arbequina*	15.74 ± 0.78^Aa^	12.47 ± 0.67^ABa^	8.30 ± 0.52^Ba^
	*Koroneiki*	33.41 ± 0.85^Ab^	30.07 ± 0.55^Ab^	23.70 ± 0.32^Bb^
	*Manzanilla*	16.25 ± 0.55^Aa^	13.77 ± 0.45^Aa^	12.25 ± 0.43^Ba^
Iodine value (g I_2_/100 g oil)	*Arbequina*	78.99 ± 0.55^Aa^	80.97 ± 0.25^Aa^	83.07 ± 0.22^ABa^
	*Koroneiki*	74.83 ± 0.15^Ab^	76.27 ± 0.20^ABb^	78.05 ± 0.40^Bb^
	*Manzanilla*	76.45 ± 0.30^Aab^	80.81 ± 0.20^Ba^	80.25 ± 0.18^ab^
Saponification value (mg KOH/g oil)	*Arbequina*	194.60 ± 0.25^Aa^	194.10 ± 0.15^Aa^	194.00 ± 0.20^Aa^
	*Koroneiki*	193.60 ± 0.11^Aa^	193.00 ± 0.24^Aa^	193.10 ± 0.30^Aa^
	*Manzanilla*	194.40 ± 0.45^Aa^	194.30 ± 0.25^Aa^	194.00 ± 0.20^Aa^

*Note*: ^A–C^represent significant differences (*p* < .05) in the same line (between different months), ^a–c^represent significant differences (*p* < .05) in the same column (between different varieties).

Results showed an increase in oil recovery percentage for each variety with developing in the ripening stage for all of the investigated types of olive fruit (*p* < .05). The highest oil content was observed in *Arbequina* during the ripening phase. The lowest oil content was obtained for *the Manzanilla* cultivar (9.35 ± 0.42%, 13.30 ± 0.48%, and 15.15 ± 0.68%) at all stages of maturation. In addition, the peroxide value for all EVOOs decreased during the maturation stages. Peroxide values of EVOOs extracted from *Arbequina* (17.56 ± 0.42 to 2.85 ± 0.16 meqO_2_/kg oil) and *Koroneiki* (17.32 ± 0.23 to 3.00 ± 0.12 meqO_2_/kg oil) cultivars were higher than *Manzanilla* cultivar (5.08 ± 0.0.18 to 1.56 ± 0.10 meqO_2_/kg oil) with developing in the ripening process of studied olive fruits. The acidity values obtained for EVOOs of *the Koroneiki* cultivar (0.17 ± 0.01% to 0.27 ± 0.04%) were higher EVOOs extracted from other studied cultivars (*Arbequina*: 0.13 ± 0.01% to 0.18 ± 0.05%, *Manzanilla*: 0.12 ± 0.04 to 0.15 ± 0.04%). Results showed some variations between acidity values during the ripening stages of fruits within and between varieties; however, these differences were not statistically significant (*p* > .05).

The amounts of oxidative stability of all EVOOs decreased with development in the ripening stages for the studied varieties of olive fruits. *Manzanilla*, *Koroneiki*, and *Arbequina* EVOOs' oxidative stability values ranged from 12.25 h to 16.25 h, 23.70 h to 33.41 h, and 8.30 h to 15.74 h, respectively. The highest oxidative stability was related to *Koroneiki* EVOOs.

The amounts of iodine value for all EVOOs increased with development in the ripening stages. Results showed that the amounts of iodine values of *Arbequina*, *Koroneiki*, and *Manzanilla* ranged from 78.99 ± 0.55 to 83.07 ± 0.22 (gI_2_/100 g oil), 74.83 ± 0.15 to 78.05 ± 0.40 (gI_2_/100 g oil), and 76.45 ± 0.30 to 80.81 ± 0.20 (gI_2_/100 g oil), respectively.

The amounts of saponification value in all olive oils showed no significant differences during the maturation stages. Results showed that the amounts of saponification value in *Arbequina*, *Koroneiki*, and *Manzanilla* ranged from 194.00 ± 0.20 to 194.60 ± 0.25 (mg KOH/g oil), 193.00 ± 0.44 to 193.60 ± 0.11(mg KOH/g oil), and 194.10 ± 0.45 to 194.60 ± 0.30 (mg KOH/g oil), respectively.

### 3.2 FAs composition

The results of FA composition are presented in Table 2. The maximum values of oleic acid belonged to the last harvest date (November) in all varieties. According to the FA composition analysis, palmitic acid levels in *Koroneiki* and *Arbequina* decreased but slightly increased in *Manzanilla* as fruit maturation advanced. The monitoring of FA composition was investigated based on parameters including different stages of maturity, the place of cultivation, and the crop season. The results showed an increase in PUFA levels as the olive maturation advanced. In addition, a decrease in MUFA level was observed in *Manzanilla* and very low in *Arbequina*, but in *Koroneiki*, an increase in MUFA level was observed. The MUFA/PUFA ratio was higher in the first (green stage) stage, decreasing as olive maturation advanced. The MUFA/PUFA ratio was higher in oils extracted from the *Koroneiki* variety than in other varieties at different ripening stages.

**TABLE 2:** FAs composition present in EVOOs analyzed, from the three olive varieties grown in the study area, collected at different stages of ripening, Data are presented as mean ± SD (*n* = 3).

FAs composition	*Arbequina*			*Koroneiki*			*Manzanilla*		
	September	October	November	September	October	November	September	October	November
C12:0	nd^a^	nd^a^	nd^a^	0.04 ± 0.00^b^	0.02 ± 0.00^c^	0.02 ± 0.00^c^	nd^a^	0.04 ± 0.02^b^	nd^a^
C14:0	0.06 ± 0.00^a^	0.03 ± 0.00^b^	0.03 ± 0.00^b^	0.01 ± 0.00^c^	0.05 ± 0.01^d^	0.03 ± 0.02^b^	0.03 ± 0.01^b^	0.03 ± 0.01^b^	0.03 ± 0.00^b^
C16:0	21.29 ± 0.08^a^	20.48 ± 0.05^a^	18.94 ± 0.08^b^	18.14 ± 0.06^b^	16.13 ± 0.09^c^	14.72 ± 0.04^d^	20.99 ± 0.03^a^	21.00 ± 0.05^a^	21.35 ± 0.04^a^
C16:1c	2.15 ± 0.03^a^	2.28 ± 0.02^a^	2.01 ± 0.02^a^	1.84 ± 0.05^a^	1.90 ± 0.06^a^	1.46 ± 0.06^b^	2.05 ± 0.01^a^	2.38 ± 0.02^c^	2.30 ± 0.01^c^
C17:0	0.13 ± 0.02^a^	0.15 ± 0.01^a^	0.12 ± 0.02^a^	0.15 ± 0.02^a^	0.03 ± 0.01^b^	0.05 ± 0.01^b^	0.04 ± 0.01^b^	0.07 ± 0.02^b^	0.12 ± 0.02^a^
C17:1c	0.22 ± 0.01^a^	0.20 ± 0.01^a^	0.15 ± 0.01^b^	0.07 ± 0.01^c^	0.11 ± 0.02^bc^	0.13 ± 0.01^b^	0.07 ± 0.01^c^	0.09 ± 0.01^c^	0.15 ± 0.02^b^
C18:0	2.24 ± 0.08^ab^	2.24 ± 0.05^ab^	2.41 ± 0.05^a^	2.39 ± 0.05	2.37 ± 0.06^a^	2.64 ± 0.07^a^	2.00 ± 0.08^b^	2.28 ± 0.05^ab^	2.42 ± 0.05^a^
C18:1c	58.24 ± 0.40^a^	58.08 ± 0.25^a^	58.55 ± 0.60^a^	70.12 ± 0.60^b^	72.17 ± 0.55^b^	72.88 ± 0.45^b^	62.65 ± 0.40^c^	56.63 ± 0.45^d^	53.24 ± 0.60^d^
C18:2c	14.18 ± 0.10^a^	15.33 ± 0.10^ab^	16.69 ± 0.10^b^	5.63 ± 0.09^c^	5.79 ± 0.10^c^	6.57 ± 0.15^c^	10.54 ± 0.10^d^	15.84 ± 0.12^ab^	16.96 ± 0.15^b^
C18:3c	0.81 ± 0.03^a^	0.60 ± 0.04^d^	0.83 ± 0.02^a^	0.90 ± 0.03^b^	0.83 ± 0.05^a^	0.99 ± 0.03^c^	0.82 ± 0.03^a^	0.87 ± 0.04^ab^	0.97 ± 0.02^c^
C20:0	0.36 ± 0.01^a^	0.28 ± 0.02^b^	0.37 ± 0.03^a^	0.34 ± 0.01^a^	0.28 ± 0.02^b^	0.36 ± 0.03^a^	0.42 ± 0.02^c^	0.49 ± 0.02^d^	0.47 ± 0.03^d^
C20:1c	0.20 ± 0.01^a^	0.21 ± 0.01^a^	0.20 ± 0.03^a^	0.26 ± 0.03^b^	0.23 ± 0.02^ab^	0.02 ± 0.03^c^	0.29 ± 0.01^d^	0.18 ± 0.01^a^	0.30 ± 0.03^d^
C22:0	nd^a^	nd^a^	0.10 ± 0.00^b^	nd^a^	nd^a^	nd^a^	nd^a^	nd^a^	0.10 ± 0.00^b^
C24:0	0.10 ± 0.01^a^	0.09 ± 0.02^a^	0.08 ± 0.02^a^	0.11 ± 0.01^a^	0.07 ± 0.02^a^	0.12 ± 0.02^a^	0.12 ± 0.01^a^	0.10 ± 0.02^a^	0.16 ± 0.01^b^
C24:1c	0.03 ± 0.01^ac^	0.03 ± 0.01^ac^	0.02 ± 0.01^ab^	0.01 ± 0.01^b^	0.03 ± 0.01^ac^	0.04 ± 0.01c	0.02 ± 0.01^ab^	0.04 ± 0.01^c^	0.02 ± 0.01^ab^
M.U.F.A.	60.84 ± 0.50^ad^	60.80 ± 0.70^ad^	60.42 ± 1.01^ad^	72.24 ± 0.65^b^	74.43 ± 0.95^b^	74.49 ± 1.02^b^	65.06 ± 0.40^c^	59.28 ± 0.70^ad^	55.99 ± 1.00^d^
P.U.F.A.	14.99 ± 0.13^a^	15.93 ± 0.50^ab^	17.52 ± 0.60^b^	6.53 ± 0.13^c^	6.62 ± 0.50^c^	7.56 ± 0.60^c^	11.36 ± 0.10^d^	16.71 ± 0.35^b^	17.93 ± 0.45^b^
MUFA/PUFA	4.06 ± 0.11^ab^	3.82 ± 0.20^a^	3.45 ± 0.18^a^	11.06 ± 0.18^c^	11.24 ± 0.20^c^	9.88 ± 0.11^c^	5.73 ± 0.20^b^	3.55 ± 0.15^a^	3.12 ± 0.15^a^
C18:1c/C18:2c	4.11 ± 0.10^a^	3.79 ± 0.13^a^	3.48 ± 0.10^a^	12.46 ± 0.30^b^	12.45 ± 0.25^b^	11.09 ± 0.20^b^	5.94 ± 0.12^c^	3.58 ± 0.11^a^	3.14 ± 0.25^a^

*Note*: ^a–d^represent significant differences in the same line (*p* < .05).

### 3.3 Sterols and triterpenes composition

Sterols composition, the main triterpenic sterols, dialcohols, and the total sterol content of each EVVO obtained from various olive varieties are presented in Table 3.

**TABLE 3:** Sterols and triterpene and dialcohol composition present in EVOOs analyzed, from the three olive varieties grown in the study area, collected at different stages of ripening, Data are presented as mean ± SD (*n* = 3).

	*Arbequina*			*Koroneiki*			*Manzanilla*		
	September	October	November	September	October	November	September	October	November
**Sterol composition**									
Cholesterol (%)	0.03 ± 0.00^a^	0.01 ± 0.00^a^	nd^a^	nd	nd	nd	0.02 ± 0.02^a^	0.09 ± 0.01^b^	0.09 ± 0.01^b^
Brassicasterol (%)	0.01 ± 0.00^a^	0.02 ± 0.00^a^	0.02 ± 0.00^a^	0.01 ± 0.00^a^	nd	nd	nd	0.03 ± 0.01^a^	0.03 ± 0.01^a^
24‐Methylene cholesterol(%)	0.05 ± 0.01^a^	0.13 ± 0.04^a^	0.02 ± 0.01^a^	0.01 ± 0.00^a^	0.10 ± 0.01^a^	0.10 ± 0.00^a^	0.04 ± 0.01^a^	0.03 ± 0.03^a^	0.01 ± 0.01^a^
Campesterol (%)	4.83 ± 0.09^a^	4.06 ± 0.12^ab^	4.08 ± 0.11^ab^	4.56 ± 0.13^a^	3.50 ± 0.20^b^	3.46 ± 0.32^b^	4.45 ± 0.08^a^	3.35 ± 0.05^b^	3.17 ± 0.10^b^
Campestanol (%)	nd	0.46 ± 0.02^a^	0.07 ± 0.02^b^	0.05 ± 0.01^b^	nd	nd	0.50 ± 0.01^a^	0.30 ± 0.02^ac^	0.23 ± 0.02^c^
Stigmasterol (%)	1.47 ± 0.03^a^	1.09 ± 0.02^b^	1.09 ± 0.01^b^	1.14 ± 0.07^b^	1.09 ± 0.05^b^	0.47 ± 0.08^c^	1.58 ± 0.05^ad^	1.73 ± 0.06^d^	1.80 ± 0.02^d^
Δ‐7‐Campestrol (%)	0.09 ± 0.01^a^	0.40 ± 0.05^b^	0.20 ± 0.02^b^	0.22 ± 0.05^b^	0.33 ± 0.07^b^	0.41 ± 0.05^b^	0.11 ± 0.08^a^	0.23 ± 0.03^b^	0.13 ± 0.02^a^
Δ‐5, 23‐Stigmastadienol (%)	0.30 ± 0.01^a^	nd	nd	0.82 ± 0.0^b^	0.09 ± 0.01^c^	0.02 ± 0.01^c^	0.18 ± 0.02^d^	0.13 ± 0.01^d^	0.05 ± 0.01^c^
Clerosterol (%)	0.98 ± 0.10^a^	0.81 ± 0.08^a^	0.40 ± 0.12^b^	0.88 ± 0.21^a^	0.65 ± 0.15^b^	0.65 ± 0.10^b^	1.31 ± 0.21^c^	0.86 ± 0.20^a^	0.55 ± 0.10^b^
β‐Sitosterol (%)	83.91 ± 0.20^ab^	82.58 ± 0.35^a^	81.04 ± 0.15^a^	85.84 ± 0.55^b^	82.43 ± 0.35^a^	81.98 ± 0.45^a^	86.73 ± 0.15^b^	83.60 ± 0.25^a^	83.16 ± 0.22^a^
Sitostanol (%)	0.38 ± 0.01^a^	0.05 ± 0.01^b^	0.01 ± 0.01^b^	0.12 ± 0.01^bc^	0.10 ± 0.02^bc^	0.10 ± 0.03^bc^	0.17 ± 0.02^c^	0.10 ± 0.03^bc^	0.04 ± 0.01^b^
Δ‐5‐Avenasterol (%)	6.24 ± 0.20^ac^	9.55 ± 0.15^b^	12.34 ± 0.30^c^	4.45 ± 0.23^c^	10.57 ± 0.44^b^	10.96 ± 0.45^bc^	4.97 ± 0.15^c^	8.41 ± 0.11^b^	9.91 ± 0.20^b^
Δ‐5, 24‐Stigmastadienol (%)	1.41 ± 0.22^a^	0.33 ± 0.02^b^	0.64 ± 0.06^c^	0.96 ± 0.01^a^	0.66 ± 0.02^c^	0.99 ± 0.03^a^	0.76 ± 0.03^d^	0.71 ± 0.02^cd^	0.19 ± 0.05^b^
Δ‐7‐Stigmastenol (%)	0.07 ± 0.01^a^	0.05 ± 0.01^a^	0.20 ± 0.03^b^	0.50 ± 0.11^b^	0.33 ± 0.15^b^	0.47 ± 0.23^b^	0.16 ± 0.01^c^	0.29 ± 0.04^b^	0.49 ± 0.08^b^
Δ‐7‐Avensterol (%)	0.23 ± 0.01^a^	0.46 ± 0.03^b^	0.29 ± 0.05^a^	0.32 ± 0.08^ab^	0.79 ± 0.04^d^	0.40 ± 0.06^b^	0.33 ± 0.02^ab^	0.14 ± 0.02^ca^	0.15 ± 0.05^ca^
Apparent β‐sitosterol (%)	92.27 ± 1.25^a^	92.47 ± 1.15^a^	94.03 ± 1.10^a^	92.19 ± 1.52^a^	93.76 ± 1.20^a^	94.05 ± 1.42^a^	92.81 ± 1.31^a^	92.95 ± 1.58^a^	93.35 ± 1.66^a^
Total Sterol (mg/kg)	1775.9 ± 0.51^a^	1617.9 ± 0.81^b^	1513.7 ± 0.55^c^	1511.61 ± 0.54^c^	1378.57 ± 0.31^d^	1091.81 ± 0.45^e^	1936.05 ± 1.31^f^	1343.5 ± 1.58^g^	1231.9 ± 1.66^h^
**Tri terpenes**									
Erythrodiol (%)	0.95 ± 0.15^a^	0.50 ± 0.03^b^	0.43 ± 0.01^b^	2.44 ± 0.50^c^	1.99 ± 0.55^ac^	1.13 ± 0.81^a^	0.36 ± 0.40^b^	0.47 ± 0.70^b^	2.47 ± 1.00^c^
Uvaol (%)	0.42 ± 0.05^a^	0.12 ± 0.01^b^	0.12 ± 0.01^b^	0.62 ± 0.13^a^	0.40 ± 0.10^a^	0.13 ± 0.09^b^	0.18 ± 0.08^b^	0.10 ± 0.01^b^	0.09 ± 0.01^b^

*Note*: ^a–h^represent significant differences in the same line (*p* < .05).

EVOOs extracted from *the Arbequina* variety had the highest values of campesterol content. In addition, the campesterol content decreased during maturation in all EVOOs. During fruit maturation, stigmasterol content decreased in EVOOs extracted from *Arbequina* and *Koroneiki* varieties but increased in *the Manzanilla* variety, and the highest stigmasterol content was observed in EVOOs obtained from *the Manzanilla* variety. The β‐sitosterol was the main sterol in all EVOOs samples, and *Manzanilla* had the highest content during each ripening stage. Also, Δ‐5‐Avenasterol content increased during fruit maturation. The total sterol content decreased for *Arbequina*, *Koroneiki*, and *Manzanilla* at different ripening stages. The sum of erythrodiol and uvaol decreased for *Arbequina* and *Koroneiki* but increased for *Manzanilla* as the olive maturity process.

### 3.4 Triglycerides composition

The determination of triacylglycerol composition is a significant factor in the classification and characterization of monovarietal olive oils. The triglyceride distribution of EVOOs is presented in Table 4. According to the results, major triglycerides were triolein (OOO) (14.082–36.394%), palmitodiolein (POO) (22.187–31.130%), dioleolinolein (OOL) (7.706–15.524%), palmitooleolinolein (PLO) (4.705–14.242%), dipalmitoolein (POP) (4.893–9.67%), stearodiolein (SOO) (2.035–4.051%). Other minor triglycerides were palmitolinolenin (PLLn) (0.055–0.263%), trilinolein (LLL) (0.017–0.466%), palmitodilinolein (PLL) (0.190–2.285%), oleolinoleolinolein (OLLn) (0.198–0.501%), dioleolinolenin (OOLn) (0.553–1.505%), oleodilinolein (OLL) (0.623–4.497%), palmitoolein (POLn) (0.746–1.215%), dipalmitoolein (POP) (4.893–8.755%), and dipalmitoolein (PoOP) (1.205–2.129%). During maturation, the OOO percentage for *Arbequina* and *Koroneiki* increased but decreased for *Manzanilla*. In addition, OOL, PLL, OLL, PLO, and SOL percentages increased during the growth of olive fruits, while POO, SOO, and POP decreased during the maturation stages.

**TABLE 4:** Triglyceride composition present in EVOOs analyzed, from the three olive varieties grown in the study area, collected at different stages of ripening.

T.A.G.s	*Arbequina*			*Koroneiki*			*Manzanilla*		
	September	October	November	September	October	November	September	October	November
LLL	0.261	0.331	0.434	0.017	0.019	0.027	0.107	0.363	0.466
PoLL	0.131	0.163	0.173	0.018	0.020	0.020	0.069	0.180	0.209
OLLn	0.367	0.294	0.450	0.201	0.198	0.273	0.297	0.427	0.501
PoOLn	0.061	0.048	0.059	0.072	0.072	0.067	0.064	0.071	0.075
PLLn	0.174	0.134	0.187	0.066	0.055	0.068	0.129	0.206	0.263
PPoLn	0.029	0.022	0.025	0.024	0.020	0.017	0.028	0.034	0.039
PoPoL	0.022	0.027	0.023	0.007	0.007	0.005	0.015	0.030	0.031
PoPoPo	0.001	0.002	0.001	0.001	0.001	0.000	0.001	0.002	0.002
SLnLn	0.001	0.000	0.000	0.001	0.001	0.001	0.000	0.001	0.001
OLL	3.186	3.734	4.497	0.623	0.686	0.901	1.897	3.862	4.352
PoOL	1.065	1.224	1.938	0.449	0.496	0.441	0.813	1.279	1.301
OOLn	0.747	0.553	0.777	1.241	1.225	1.505	0.877	0.758	0.781
PLL	1.515	1.700	1.868	0.204	0.190	0.223	0.822	1.858	2.285
POLn	1.053	0.746	0.956	1.199	1.004	1.104	1.126	1.080	1.215
PPoPo	1.421	1.475	0.984	0.757	0.634	0.312	1.250	1.686	1.748
PoOO	2.170	2.300	2.061	2.776	3.070	2.429	2.399	2.269	2.027
PoPoO	0.089	0.100	0.079	0.081	0.090	0.054	0.087	0.106	0.097
PPoL	0.506	0.557	0.496	0.147	0.138	0.109	0.353	0.616	0.683
OOL	12.988	14.032	15.524	7.706	8.487	9.916	11.193	13.706	13.559
PoOO	2.170	2.300	2.061	2.776	3.069	2.429	2.399	2.269	2.027
SLL	0.144	0.168	0.214	0.024	0.025	0.036	0.071	0.182	0.233
PLO	12.355	12.777	12.898	5.035	4.705	4.920	9.704	13.188	14.242
PLP	2.148	2.125	1.955	0.600	0.475	0.444	1.537	2.318	2.734
PoPP	0.378	0.367	0.274	0.228	0.182	0.115	0.347	0.404	0.429
PoOP	2.065	2.094	1.712	1.814	1.702	1.205	2.080	2.184	2.129
LnPP	2.130	0.088	0.103	0.102	0.073	0.072	0.127	0.135	0.166
SPoL	0.048	0.055	0.057	0.017	0.018	0.018	0.030	0.060	0.070
SOLn	0.067	0.050	0.074	0.095	0.091	0.121	0.065	0.071	0.083
OOO	17.648	17.577	18.864	31.762	35.008	36.394	22.011	16.212	14.082
POO	25.183	24.007	22.261	31.130	29.112	27.088	28.627	23.399	22.187
POP	8.755	7.984	6.749	7.419	5.879	4.893	9.670	8.227	8.519
SLL	0.144	0.168	0.214	0.024	0.025	0.036	0.071	0.182	0.234
PPoO	2.065	2.095	1.712	1.814	1.702	1.205	2.080	2.184	2.129
PLS	0.825	0.849	0.909	0.287	0.258	0.292	0.535	0.919	1.132
PoPP	0.378	0.370	0.274	0.228	0.182	0.115	0.347	0.404	0.429
SOL	1.157	1.240	1.459	0.585	0.619	0.784	0.823	1.274	1.436
SOO	3.031	2.340	2.035	4.051	4.050	3.065	4.032	3.380	3.029
POS	1.747	1.658	1.633	1.845	1.662	1.676	1.639	1.694	1.830
SLS	0.204	0.209	0.224	0.071	0.063	0.072	0.132	0.227	0.279

Abbreviations: L, linoleic acids; O, oleic; P, palmitic; Po, palmitoleic; S, stearic.

### 3.5 Total aliphatic alcohols

Results showed that the total aliphatic alcohol contents of EVOO varied significantly based on the variety (*p* < .05) (Table 5). The total aliphatic alcohol contents were from 780.07 ± 0.20 to 411.24 ± 0.45 mg/kg for *Arbequina*, 937.70 ± 0.44 to 386.15 ± 0.44 mg/kg for *Koroneiki*, and 1097.67 ± 0.18 to 286.10 ± 0.50 mg/kg for *Manzanilla*. The total aliphatic alcohol contents of all cultivars significantly decreased with the ripening of olive fruit. The total aliphatic alcohol contents of studied EVOOs were affected considerably by ripening stages (*p* < .05).

**TABLE 5:** Total aliphatic alcohols content of EVOOs from three olive varieties at different stages of ripening.

Sample name	September	October	November
*Arbequina* (mg/kg oil)	1780.07 ± 0.20^Aa^	1136.18 ± 0.25^Ba^	411.24 ± 0.45^Ca^
*Koroneiki* (mg/kg oil)	937.70 ± 0.44^Ab^	711.87 ± 0.55^Bb^	386.15 ± 0.44^Cb^
*Manzanilla* (mg/kg oil)	1097.67 ± 0.18^Ac^	743.06 ± 0.33^Bc^	286.10 ± 0.50^Cc^

*Note*: Data are presented as mean ± SD (*n* = 3). ^A–C^represent significant differences (*p* < .05) in the same line (between different months), ^a–c^represent significant differences (*p* < .05) in the same column (between different varieties).

### 3.6 Polyphenols composition

Ripening of olive fruit influenced the phenolic contents of the EVOOs. The value of polyphenol content of extracted oils from green olive fruits was higher than from ripe olive fruits. Results showed that oleuropein decreased while the oleuropein aglycone, oxidized aldehyde, and hydroxylic form increased at different ripening stages for all EVOOs (Table 6). In addition, decarboxymethyl oleuropein aglycone and dialdehyde form decreased in *Arbequina* and *Manzanilla* cultivars while rising in the *Koroneiki* cultivar. Oleuropein aglycone, aldehyde, and hydroxylic form increased in *Arbequina* and *Manzanilla* though decreased in *Koroneiki*. The amounts of apigenin, quercetin, ferulic acid, caffeic acid, and catechin decreased during the maturation of fruits. For all EVOOs, the amounts of hydroxytyrosol and tyrosol increased through different ripening stages of fruits.

**TABLE 6:** Individual phenolic compounds of EVOOs obtained from three olive varieties at different stages of ripening.

Major phenols composition (%)	*Arbequina*			*Koroneiki*			*Manzanilla*		
	September	October	November	September	October	November	September	October	November
Gallic acid	2.10 ± 0.13^a^	2.40 ± 0.18^ad^	2.50 ± 0.20^ad^	1.04 ± 0.12^b^	0.88 ± 0.15^b^	0.52 ± 0.11^b^	5.10 ± 0.10^c^	4.05 ± 0.25^cd^	3.90 ± 0.35^cd^
Hydroxytyrosol	0.50 ± 0.11^a^	1.53 ± 0.35^ab^	3.00 ± 0.37^b^	1.01 ± 0.08^a^	2.55 ± 0.10^b^	3.45 ± 0.89^b^	0.60 ± 0.01^a^	0.89 ± 0.04^a^	1.35 ± 0.24^a^
Tyrosol	0.90 ± 0.08^a^	1.48 ± 0.05^b^	3.94 ± 0.09^c^	1.14 ± 0.06^b^	3.50 ± 0.09^c^	5.72 ± 0.04^d^	5.99 ± 0.88^d^	6.56 ± 0.67^d^	7.35 ± 0.55^d^
Catechin	1.00 ± 0.03^ac^	0.88 ± 0.02^a^	0.75 ± 0.05^a^	1.84 ± 0.05^bc^	1.20 ± 0.30^c^	1.16 ± 0.20^c^	3.35 ± 0.45^d^	2.38 ± 0.55^cd^	1.30 ± 0.33^c^
Caffeic acid	1.50 ± 0.02^a^	1.15 ± 0.01^ab^	1.12 ± 0.02^ab^	2.70 ± 0.22^a^	1.65 ± 0.15^a^	1.05 ± 0.01^b^	3.89 ± 0.34^a^	2.56 ± 0.67^a^	1.12 ± 0.34^ab^
Vanillin	1.22 ± 0.01^a^	1.20 ± 0.05^a^	1.00 ± 0.01^a^	0.87 ± 0.02^a^	2.11 ± 0.22^b^	5.56 ± 0.33^c^	2.07 ± 0.44^a^	5.09 ± 0.55^c^	6.15 ± 0.78^c^
Vanillic acid	2.24 ± 0.08^a^	2.24 ± 0.05^a^	2.41 ± 0.05^a^	1.39 ± 0.44^a^	2.66 ± 0.23^a^	5.64 ± 0.77^b^	4.00 ± 0.23^b^	3.28 ± 0.55^ab^	2.42 ± 0.25^a^
p‐Coumaric acid	1.00 ± 0.40^a^	2.18 ± 0.25^a^	3.05 ± 0.60^a^	1.88 ± 0.60^a^	1.17 ± 0.55^a^	1.00 ± 0.45^a^	1.90 ± 0.40^a^	1.63 ± 0.24^a^	1.24 ± 0.14^a^
Ferulic acid	4.98 ± 0.12^a^	2.33 ± 0.10^b^	2.00 ± 0.21^b^	1.63 ± 0.09^b^	1.46 ± 0.10^b^	0.57 ± 0.15^b^	3.54 ± 0.10^c^	3.00 ± 0.12^c^	2.96 ± 0.05^c^
Decarboxymethyl oleuropein aglycone, dialdehyde form	4.81 ± 0.03^a^	2.60 ± 0.04^b^	1.83 ± 0.02^b^	3.90 ± 0.54^c^	5.83 ± 0.66^cd^	6.99 ± 0.47^cd^	16.82 ± 1.78^e^	15.87 ± 1.04^e^	11.97 ± 0.33^f^
Oleuropein	5.80 ± 0.89^a^	3.28 ± 0.45^a^	1.37 ± 0.23^b^	2.34 ± 0.23^ba^	1.28 ± 0.12^b^	0.98 ± 0.15^b^	5.42 ± 0.45^a^	4.49 ± 0.49^a^	3.47 ± 0.78^ab^
Oleuropein aglycone, oxidized aldehyde and hydroxylic form	17.80 ± 0.89^a^	21.28 ± 0.45^a^	25.37 ± 0.23^b^	10.34 ± 0.14^c^	15.28 ± 0.10^a^	19.98 ± 0.24^a^	14.34 ± 2.23^ac^	17.28 ± 1.12^a^	21.98 ± 2.15^ab^
Cinnamic acid	3.80 ± 0.22^a^	3.28 ± 0.45^a^	3.00 ± 0.23^a^	2.34 ± 0.23^ab^	1.45 ± 0.12^b^	0.98 ± 0.15^b^	4.34 ± 0.88^ca^	5.28 ± 0.98^c^	6.98 ± 0.88^c^
Quercetin	10.20 ± 0.01^a^	9.21 ± 0.01^a^	7.20 ± 0.03^b^	5.26 ± 0.17^c^	4.23 ± 0.13^c^	2.02 ± 0.33^e^	1.50 ± 0.22^e^	1.18 ± 0.15^e^	1.00 ± 0.12^e^
Luteolin	2.00 ± 0.034^ab^	2.50 ± 0.11^a^	0.98 ± 0.11^b^	6.45 ± 0.21^c^	5.00 ± 0.34^c^	2.25 ± 0.18^a^	1.40 ± 0.00^ab^	2.88 ± 0.40^a^	4.10 ± 0.55^c^
Oleuropein aglycone, aldehyde and hydroxylic form	10.10 ± 0.13^a^	14.09 ± 0.02^b^	17.08 ± 0.02^c^	24.11 ± 1.9^d^	22.88 ± 0.66^d^	20.12 ± 0.89^d^	0.99 ± 0.33^e^	2.99 ± 0.22^ef^	4.16 ± 0.45^f^
Apigenin	2.55 ± 0.21^a^	1.88 ± 0.15^ab^	1.02 ± 0.22^b^	4.01 ± 0.34^c^	3.55 ± 0.44^c^	2.04 ± 0.65^ab^	3.50 ± 0.87^c^	2.54 ± 0.67^a^	2.02 ± 0.66^ab^
Methyl‐Luteolin	5.84 ± 0.50^a^	5.60 ± 0.70^a^	3.42 ± 1.01^a^	4.24 ± 0.65^a^	3.43 ± 0.95^a^	1.49 ± 0.99^ab^	0.67 ± 0.12^b^	0.28 ± 0.15^c^	0.12 ± 0.10^c^
Ligstroside aglycone, dialdehyde form	9.99 ± 0.13^a^	5.93 ± 0.50^b^	3.52 ± 0.60^c^	6.53 ± 0.13^b^	5.62 ± 0.50^b^	2.56 ± 0.60^c^	5.67 ± 0.40^b^	4.28 ± 0.70^c^	2.99 ± 0.89^c^
Others	14.67 ± 1.13^a^	14.96 ± 2.21^a^	15.44 ± 2.43^a^	16.98 ± 2.13^a^	14.27 ± 2.21^a^	15.92 ± 1.43^a^	14.91 ± 1.45^a^	13.49 ± 2.00^a^	13.44 ± 1.66^a^

*Note*: Data are presented as mean ± SD (*n* = 3). ^a–f^represent significant differences in the same line (*p* < .05).

## 4 DISCUSSION

Finding the optimum harvesting time for obtaining the maximum yield and high‐quality EVOO is a critical parameter. The maturation of olives was associated with several physiological and chemical changes in olive fruit. Therefore, the maturity stage is an essential factor affecting olive oil composition. The level of oil extractability from olive fruit is an essential factor that influences the determination of optimum harvesting time. The rise of oil content in November might be due to the decrease in the moisture level of the extracted oil during the late maturation stage. The results of the oil content of the *Manzanilla* variety in different stages of maturity cultivated in Egypt ranged from 15.84 ± 0.36% to 25.76 ± 0.49%, while in our study, the oil contents of *Manzanilla* (9.35 to 15.15%) were lower than the data reported by Sohaimy et al. (2016). It was found that the wet oil content from green to spotted stages of olive fruit maturation for all studied varieties of the southwest of Spain (*Arbequina*, *Corniche*, *Morisca*, *Cacerena*, *Carrasquena*, *Manzanilla*, *Picual*, *Morisca*, and *Verda de Badojoz*) (Franco et al., 2015) agrees with results of this study.

The decrease in peroxide values was observed during olive ripening; therefore, EVVOs obtained from olive fruits at the more advanced stages of maturity had lower peroxide values due to decreased lipoxygenase activity. These results agreed with data reported by Youssef et al. (2010). In addition, none of the olive oil samples exceeded the maximum peroxide value specified (20 meq O_2_/kg) for EVOO in the international standard (COI/T.15/NC No 3/Rev. 17, 2021). There were statistical differences between peroxide values during the ripening of fruits (*p* < .05).

Results showed that the acidity values of none of the extracted olive oils from three studied cultivars exceeded the specified value of 0.8 (%m/m expressed in oleic acid) for EVOO in international standard COI/T.15/NC No 3/Rev. 17 (2021). Olive fruits at later stages of maturity yield olive oils with higher amounts of free acidity because of the increase in the enzymatic activity, particularly by lipolytic enzymes. Differences in olive fruit maturity might describe differences between the acidity values of various olive oils. Therefore, a high level of acidity value and increase in free fatty acids content can be related to the advanced state of fruit ripeness and the action of lipase enzyme on the olive oil triglycerides (Pérez et al., 2021). Boussahel et al. (2020) analyzed the peroxide value and acidity value in five olive oil varieties from northeast Algeria and obtained 12.75 to 15.50 meqO_2_/kg oil and 0.48 ± 0.03%–1.25 ± 0.11%, respectively (Boussahel et al., 2020). According to the international standard COI/T.15/NC No 3/Rev. 17 (2021), the primary olive oil classification is based on acidity value. Regarding the results of the acidity and peroxide values for the studied cultivars, all olive oils extracted during the three stages of harvesting were classified as extra virgin olive oils (EVOOs).

There is no specified limitation for oxidative stability by the international standard COI/T.15/NC No 3/Rev. 17 (2021), but the results of this study showed that all EVOOs had the appropriate oxidative stability. The highest oxidative stability was observed in *Koroneiki* variety EVOOs, higher than many vegetable oils. This high oxidative stability might be due to the high amounts of polyphenol compounds in the EVOOs of the *Koroneiki* variety. In addition, results showed that the amount of oxidative stability depended on the variety and maturation of olive fruits, which can be attributed to the polyphenol and oleic acid content present in the extracted olive oil.

Also, a higher iodine value shows a higher degree of unsaturation in fat or vegetable oil, indicating the oil's oxidation stability (Abril et al., 2019). In a study by Sohaimy et al., the iodine value of *Manzanilla* oil was obtained from 74.32 ± 1.4 to 90.04 ± 1.33 mgI_2_/g oil (Sohaimy et al., 2016). In the present study, the iodine value was from 76.45 ± 0.30 to 80.25 ± 0.18 mgI_2_/g oil. The saponification value depends on several factors, including pressing conditions, cultivar, climate, altitude, and geographical variations (Sohaimy et al., 2016). The present study showed no significant difference between saponification values during the ripening stages of all three studied cultivars (*p* < .05). In addition, there are no specified limitations for saponification and iodine values in the international standard COI/T.15/NC No 3/Rev. 17 (2021).

The maximum values of oleic acid were related to the last harvest date (November) in all varieties reported by other studies (Douzane et al., 2012; Faci et al., 2021). The FA composition of olive oils is affected by various parameters, mainly the olive variety, climate, the growing Area, and the ripening stage at which the fruit olives are harvested (Mena et al., 2018). The main FAs were oleic, palmitic, and linoleic acids, respectively (Mena et al., 2018). The high oleic acid content of olive oil is linked to specific properties such as an increase in oxidative stability, a decrease in low‐density lipoprotein (LDL), and antihypertensive activity (Mena et al., 2018). It was reported that olive oil samples extracted from the *Koroneiki* variety cultivated in *Messinia*, *Peloponnesus*, Greece, did not significantly differ in oleic/linoleic acid between the first and last harvest. However, there was a decreasing trend of oleic/linoleic acids during the maturation steps. The present study showed a significant difference between the first and last harvest, which agrees with other studies (Anastasopoulos et al., 2011). In addition, the oleic /linoleic acids, the MUFA/PUFA, and unsaturated fatty acid/saturated fatty acid ratios of olive oils extracted from *Castellana* in three consecutive crop seasons from four different olive‐growing regions of Madrid remain stable during fruit maturation (Mena et al., 2018), which are not in agreement with the present study. Furthermore, wide variations and important differences among fatty acid profiles of three cultivars (*Moroccan Picholine*, *Languedoc Picholine*, and *Frantoio*) grown in Errachidia (East Morocco) were reported during the two crop seasons in the different stages of maturation (Qarnifa et al., 2019).

Sterols are the main constituents of unsaponifiable matter of olive oils, and their Determination is necessary for olive oil adulteration detection and authenticity checking. The sterol composition determination can be applied to classifying virgin olive oils based on the fruit variety. The total sterol content of olive oils can be affected by crop year, geographic factors, fruit ripeness, type, and storage time before oil extraction. Yorulmaz et al. (2013) reported that the total sterol content of *Memecik* VOO decreased from 1747.47 to 1479.28 mg/Kg during the ripening stage, and β‐sitosterol had the same decreasing trend with total sterols, and the same results were obtained in our studied varieties. Although Mena et al. (2018) reported that all the sterols and total sterol content of *Castellana* olive oils were stable during the olive ripening stages, these results do not agree with the data reported by the present study.

The triterpene alcohols such as erythrodiol and uvaol are part of the unsaponifiable matter of olive oil. These triterpenes are usually determined with the sterol fraction and mainly exist in the exocarp of olive fruit (Cano et al., 2016). The EU regulatory limit for the sum of erythrodiol and uvaol content was 4.5% for the category of EVOO. For all mentioned EVOOs in the present study, the sum of erythrodiol and uvaol was lower than 4.5% specified in the COI/T.15/NC No 3/Rev. 17 (2021). Boulkroune et al. (2017) reported a downward trend in the amounts of triterpenic diols when the ripeness index increased which in our study, triterpenic diols (erythrodiol and uvaol) decreased for *Koroneiki* and *Arbequina* cultivars but increased for *Manzanilla* cultivar during maturation stages (Boulkroune et al., 2017). It was noticed that the maturity stages had an essential impact on the quantity of triterpenic dialcohols, and these results agree with the results of Mena et al. (2018) and Yorulmaz et al. (2013).

The Oleuropein content of olive fruits is attributed to the β‐glucosidase activity, which transforms oleuropein into aglycones which may be affected by the olive fruit cultivar (Damtoft et al., 1993; Fratianni et al., 2019).

Yorulmaz et al. (2013) reported that differences in the phenolic composition of varieties during the maturity stage are attributed to different phenolic compound metabolisms, cultivars, and geographical origination. It was found that during maturation, the phenolic composition of olive oil significantly changed due to the enzymatic activity. In addition, oleuropein content reaches the lowest level in overripe olive fruits (Mena et al., 2018; Yorulmaz et al., 2013). Also, a decrease in phenolic content during maturation was reported by other authors (Mena et al., 2018; Qarnifa et al., 2019; Yorulmaz et al., 2013), which is attributed to the polyphenol oxidase activity and the presence of holes exposed the pulp of olive to environmental parameters. Therefore, the harvest of olive fruit at the first stages of ripening is recommended due to the highest polyphenol content (Mena et al., 2018; Qarnifa et al., 2019).

Yorulmaz et al. (2013) reported that the OOO was the main triglyceride, between 36.58 and 39.06% for *Memecik* and between 37.06 and 38.12% for *Edremit*. In addition, the amount of OOO decreased as the ripening proceeded for *Memecik* and *Edremit*, while, in our study, OOO value increased for *Arbequina* and *Koroneiki* cultivars but decreased for *the Manzanilla* cultivar during maturation. Likewise, the same results were reported by Baccouri et al. (2008) for Tunisian VOOs during ripening. The OOP decreased while OOL and PLO increased as maturation proceeded for *Memecik* and *Edremit* varieties (Yorulmaz et al., 2013).

Pardo et al. (2020) reported that the highest stability was found in virgin olive oils containing the highest polyphenol content; therefore, the oxidative stability of virgin olive oils directly depends on the total polyphenol content. Kafkaletou et al. (2021) reported that phenolic compounds decreased during olive fruit development on the tree. Also, tyrosol is present at lower levels than hydroxytyrosol. During the olive maturation process, qualitative and quantitative changes in phenolic content happen, and these variations are attributed to a series of enzymatic and chemical alterations of some phenolic compounds (Kafkaletou et al., 2021). In addition, environmental factors and cultivation practices could influence the phenolic compounds in olives and olive products (Anastasopoulos et al., 2011).

The high content of total aliphatic alcohols in olive oils is attributed to the free form of aliphatic alcohols rather than waxes. The high level of free form of aliphatic alcohols is related to adverse climatic conditions (Boulkroune et al., 2017). Boulkroune et al. (2017) reported that the total aliphatic alcohol contents were severely affected by the maturation process and decreased significantly during the ripening stages. Our findings agree with the findings of Boulkroune et al. (2017).

## 5 CONCLUSIONS

The present study provides information about three olive cultivars, including *Arbequina*, *Koroneiki*, and *Manzanilla*, cultivated in Rudbar County, Gilan province, Iran, and how their maturation can affect extracted extra virgin olive oils' physicochemical properties and polyphenol contents.

Results showed that by progress in maturity, their oil content, acidity value, and iodine value increased, while peroxide value, oxidative stability, saponification value, total aliphatic alcohols, and unsaponifiable matter decreased. In addition, PUFA increased, and MUFA/PUFA ratio decreased during the olive maturation in all EVOOs extracted from the three examined varieties. Total sterol content and total aliphatic alcohol content decreased as maturity stages proceeded. Also, during the ripening stages, triolein (OOO), palmitodiolein (POO), dioleolinolein (OOL), and palmitooleolinolein (PLO) were the predominant triglycerides in all studied EVOOs. Oleuropein aglycone, oxidized aldehyde, and hydroxylic form, hydroxytyrosol, and tyrosol increased, but oleuropein, apigenin, quercetin, ligstroside aglycone, aldehyde and hydroxylic form, ferulic acid, caffeic acid, and catechin decreased through different ripening stages for all EVOOs. This research evidenced that maturation significantly influences the chemical composition and polyphenol contents of EVOOs.

## AUTHOR CONTRIBUTIONS

**Seyed Amirreza Ghreishi Rad:** Formal analysis (equal). **Farzaneh Ansari:** Writing – original draft (supporting).

## FUNDING INFORMATION

All authors declare that no funds and grants were received during this research.

## CONFLICT OF INTEREST STATEMENT

There are no conflicts of interest concerning the research described in this manuscript.

## Data Availability

The current study is available from the corresponding author upon reasonable request.
